# Universal optical conductivity of a disordered Weyl semimetal

**DOI:** 10.1038/srep32446

**Published:** 2016-08-30

**Authors:** Bitan Roy, Vladimir Juričić, Sankar Das Sarma

**Affiliations:** 1 Condensed Matter Theory Center and Joint Quantum Institute, University of Maryland, College Park, Maryland 20742-4111, USA; 2 Nordita, Center for Quantum Materials, KTH Royal Institute of Technology and Stockholm University, Roslagstullsbacken 23, 10691 Stockholm, Sweden

## Abstract

Topological Weyl semimetals, besides manifesting chiral anomaly, can also accommodate a disorder-driven unconventional quantum phase transition into a metallic phase. A fundamentally and practically important question in this regard concerns an experimentally measurable quantity that can clearly distinguish these two phases. We show that the optical conductivity while serving this purpose can also play the role of a bonafide order parameter across such disorder-driven semimetal-metal quantum phase transition by virtue of displaying distinct scaling behavior in the semimetallic and metallic phases, as well as inside the quantum critical fan supporting a non-Fermi liquid. We demonstrate that the correction to the dielectric constant and optical conductivity in a dirty Weyl semimetal due to weak disorder is independent of the actual nature of point-like impurity scatterers. Therefore, optical conductivity can be used as an experimentally measurable quantity to study the critical properties and to pin the universality class of the disorder-driven quantum phase transition in Weyl semimetals.

Understanding and characterizing phase transitions is one of the most important problems in condensed matter physics. Identification of distinct phases of matter and the possible phase transitions among them necessarily rely on the existence of a physical quantity that behaves differently in two phases and as such can potentially serve as a bonafide *order parameter* (OP) across the transition. The notion of an OP is as germane near a zero-temperature quantum phase transition (QPT), driven by quantum fluctuations, as near the finite-temperature classical phase transition, where thermal fluctuations dominate^1^,^2^. With increasing complexity of various phases, the horizon of OPs has expanded enormously, and topological OPs, which globally characterize a phase of matter, have recently emerged^3^,^4^. Moreover, the landscape of topological states has been extended to gapless systems featuring quasiparticles at arbitrarily low energies in the bulk, with Weyl semimetal (WSM), discovered in various three-dimensional gapless semiconductors^5^,^6^,^7^,^8^,^9^,^10^,^11^,^12^,^13^,^14^, standing as the paradigmatic representative. The constituting Weyl nodes are topologically protected and act as a source (monopole) and a sink (anti-monopole) of Berry flux in the momentum space, manifesting through Adler-Bell-Jackiw chiral anomaly and surface Fermi arcs^15^,^16^.

In addition to its topological properties, WSM can also support a disorder-tuned unconventional QPT toward a diffusive metallic phase at a finite disorder strength^17^,^18^,^19^,^20^,^21^,^22^,^23^,^24^,^25^,^26^,^27^,^28^,^29^,^30^,^31^,^32^, see Fig. 1, and we propose that optical conductivity (OC) can expose the rich phase diagram of a dirty WSM at finite frequencies. Unveiling such novel quantum critical phenomena in real materials, however, necessarily encounters technical difficulties. For example, the average density of states at the Weyl points, although has been proposed as a possible OP across such semimetal-metal QPT^25^,^27^,^29^,^30^,^31^, its measurement through compressibility in three-dimensional systems is extremely challenging, and may become even more complicated due to unwanted but likely presence of charged puddles^33^,^34^, Lifshitz tail and rare region effects^35^,^36^ in vicinity of the Weyl nodes, as well as due to pinning of the Fermi energy away from the Weyl points. These mechanisms can musk the WSM-metal quantum critical point (QCP)^33^,^34^ or perhaps even convert it into a hidden QCP^35^,^36^, therefore demanding the search of a measurable quantity that can unearth the underlying QCP by exposing the wide quantum-critical regime away from the pristine QCP [see Fig. 1]. While its inter-band component is capable of bypassing these barriers, we show here that the OC can also display a single parameter scaling across the WSM-metal QPT, thus being suitable as a promising candidate for an experimentally viable OP in a dirty WSM.

We establish that while the OC vanishes linearly with frequency (Ω) in a clean WSM, weak disorder leads to a nontrivial but *universal* (up to a sign) correction, irrespective of the actual nature of elastic scatterers. Thus both clean and weakly disordered WSMs behave as *power-law insulators*. It is worth mentioning that OC (*σ*) has been experimentally measured in the three-dimensional materials featuring linearly dispersing quasiparticles in the bulk, suggesting that *σ*(Ω) ~ Ω^37^,^38^,^39^. On the other hand, in the metallic phase the zero-frequency OC becomes finite, displaying a universal power-law dependence on disorder strength (measured from the critical one), set only by the correlation length exponent (*ν*). Inside the quantum critical regime, constituted by disorder-induced strongly coupled gapless critical modes supporting a non-Fermi liquid, the OC vanishes as *σ*(Ω) ~ Ω^1/*z*^ when frequency Ω → 0, with *z* being the dynamic scaling exponent that together with correlation length exponent (*ν*) defines the universality class of the WSM-metal QPT. As we show here, measurement of OC in a wide frequency range in fact offers a unique opportunity to unearth the universality class of the WSM-metal QPT, besides exposing a rich phase diagram of dirty WSMs. Although we here analyze the scaling behavior of OC at *T* = 0, its jurisdiction covers the entire *collisionless* regime 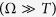.

## Results

### Model

Quintessential properties of a WSM can be captured by the effective low-energy Hamiltonian


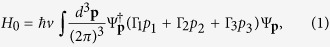


where *v* is the the Fermi velocity of Weyl fermions, assumed here to be isotropic for simplicity, and *p*_*j*_ are components of momentum. Three mutually anticommuting matrices are defined as Γ_*j*_ = *τ*_3_ ⊗ *α*_*j*_ with two sets of Pauli matrices *τ* and *α* respectively acting in the chiral (valley) and spin spaces. The spinor is defined as 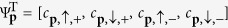, where *c*_**p**,*σ*,*τ*_ is the fermion annihilation operator with momentum **p** (measured from the Weyl nodes), spin projection *α* = ↑/↓, and chirality *τ* = +/− (left/right). As shown in the Supplementary Information (SI), the above low-energy Hamiltonian for WSM can be realized from a simple tight-binding model on a cubic lattice. Integrals over momentum run up to an ultraviolet cutoff Λ ~ 1/*a*, with *a* being the lattice spacing. The above Hamiltonian enjoys a global chiral U(1) symmetry, generated by *γ*_5_ = *τ*_3_ ⊗ *α*_0_, which in the continuum limit also stands as the generator of translational symmetry^40^.

### Disorder

Weyl fermions are susceptible to various disorder and the scattering processes by different types of impurities^41^, represented by potential terms coupled to appropriate fermion bilinears. Effects of randomness are captured by the Euclidean action 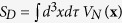 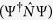, where *V*_*N*_(**x**) for simplicity assumes a Gaussian white noise distribution, with disorder average 〈〈*V*_*N*_(**x**)*V*_*N*_(**x**′)〉〉 = Δ_*N*_*δ*(**x** − **x**′). As shown in the SI, various types of disorder can be described by an appropriate choice of the 4 × 4 matrix 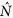 and the scaling dimension of disorder coupling [Δ_*N*_] = 2*z* − *d*. The clean WSM features linearly dispersing quasiparticles and is thus characterized by dynamic scaling exponent *z* = 1. Therefore, sufficiently weak disorder is an *irrelevant* perturbation, since [Δ_*N*_] = −1, and low energy excitations retain their ballistic nature in weakly disordered WSMs.

The fact that weak disorder is an irrelevant perturbation in three-dimensional WSM gives rise to the possibility of a disorder-driven QPT to a metallic phase for strong disorder^17^,^18^,^19^,^20^,^21^,^22^,^23^,^24^,^25^,^26^,^27^,^28^,^29^,^30^,^31^,^32^. In light of this, we next show that OC exhibits a single parameter scaling, and therefore can serve as a bonafide OP across the WSM-metal QPT. For extremely strong disorder, the three-dimensional metal eventually undergoes the *Anderson transition* into an insulating phase^17^,^27^, which is, however, outside the scope of the current work.

### Scaling

**Figure 1:**
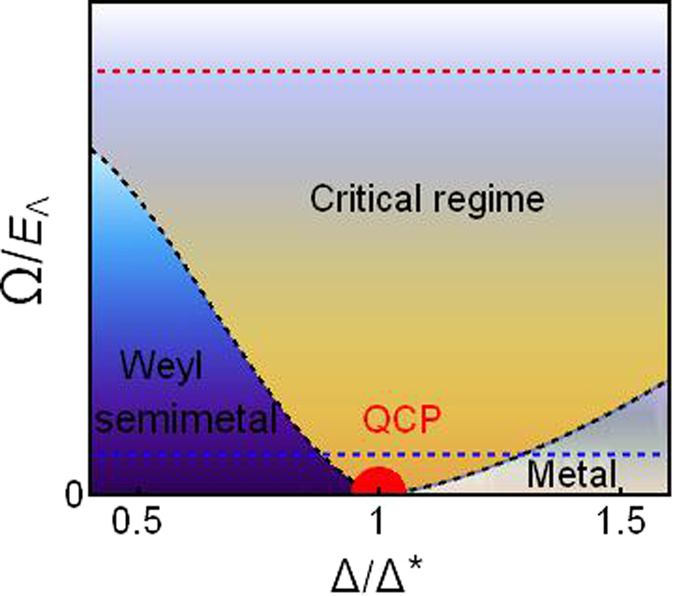
A schematic phase diagram of a dirty Weyl semimetal at finite frequencies (Ω), subject to random charge impurities, where *E*_Λ_ ~ *v*Λ is the ultraviolet cutoff for energy. All the phases and the quantum critical point (red dot) exist only at zero frequency. Various crossover boundaries (black dashed lines), such as the ones between the critical regime and Weyl semimetal or metal, have been estimated from the scaling of specific heat at finite temperatures^29^ and average density of states at finite energies^31^. The red line marks the high energy cut-off above which the continuum description of a WSM based on linearly dispersing quasiparticles breaks down. Blue line shows the location of Fermi energy (often unknown). WSM-metal QPT is tuned by disorder (Δ) and takes place at a critical strength of disorder Δ = Δ* (see text). The optical conductivity inside the Weyl semimetal and critical regime respectively scales/vanishes as Ω and Ω^1/*z*^, while it becomes finite in the metallic phase as Ω → 0. As frequency is increased optical conductivity displays smooth crossovers between distinct regimes (represented by color gradient in the phase diagram). In the vicinity of the WSM-metal QCP at Δ = Δ*, the phase boundary between the critical regime and WSM or metal scales as *δ*^*νz*^ (see text). In the plot we use the one-loop result for the critical exponents *ν* = 1 and *z* = 3/2 at the QCP corresponding to the QPT driven by the potential or axial chemical potential disorder.

The scaling of conductivity (*σ*) with the system size (*L*) follows from the gauge invariance, leading to *σ* ~ *L*^2−*d*^, see SI. As the system approaches the QCP located at 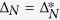, the correlation length (*ξ*) diverges according to *ξ* ~ |*δ*|^−*ν*^, while the corresponding energy (*ε*_0_) vanishes as *ε*_0_ ~ |*δ*|^*νz*^, where 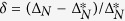 measures the distance from the QCP. Therefore, semimetallic and metallic phases are respectively realized for 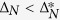 and 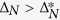. In the proximity to a QCP, the universal scaling of any physical observable depends on two dimensionless parameters *L*/*ξ* and Ω/*ε*_0_. Thus general scaling theory and gauge invariance dictate the following scaling ansatz for the OC (in units of *e*^2^/*h*) in a dirty WSM


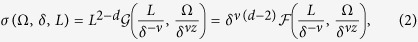


where 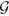 and 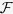 are two unknown, but universal scaling functions. Although the explicit forms for these scaling functions are generally unknown and can only be determined experimentally, their salient features can be deduced from the behavior of OC in various phases of a dirty WSM. Since we are interested in the optical properties of a WSM in the thermodynamic limit (*L* → ∞), for brevity we drop the explicit *L*-dependence in *σ*(Ω, *δ*, *L*). Although we here exploit the gauge invariance and scaling theory to obtain the scaling ansatz in Eq. (2), this can also be achieved from the renormalization group analysis of the disorder coupling, as shown in the SI. When the Fermi energy (*E*_*F*_) is pinned away from the Weyl points (see red dashed line in Fig. 1), the system behaves as a diffusive metal at the lowest energy scale for arbitrary strength of impurity scatterers and our discussion on the scaling of OC is germane only for Ω > *E*_*F*_.

First we focus on the QCP (*δ* = 0), where the OC must be devoid of any *δ*-dependence, dictating 
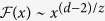
. Its scaling with frequency is then given by
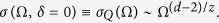


Therefore, within the critical regime OC vanishes with a peculiar power-law dependence when frequency Ω > *ε*_0_ ~ |*δ*|^*νz*^, which in turn roughly determines the extent of the critical regime at finite frequencies (see Fig. 1). Notice that as the QCP is approached from the WSM phase the residue of quasiparticle pole vanishes smoothly^21^, while approaching it from the metallic side the diffusion coefficient diverges^25^. Therefore, the critical regime constitutes a *non-Fermi liquid* phase of strongly coupled gapless critical modes, due to quantum fluctuations driven by disorder, where the OC scales as Ω^1/*z*^.

Next we consider the metallic phase, where average density of states at zero energy is finite, and thus OC as Ω → 0 also becomes finite due to a finite lifetime of diffusive fermions. Hence, inside the metallic phase 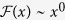 (to the leading order) and OC scales as


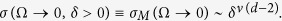


OC in the metallic phase thus depends only on *ν* as Ω → 0, which together with the dependence of *σ*_*Q*_(Ω) solely on *z* endows a unique opportunity to extract the correlation length and the dynamic critical exponents near the WSM-metal QCP independently, and that way pin the universality class of this transition. Hence, in the presence of strong disorder 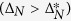, as the frequency is gradually lowered the intra-band component of OC starts to dominate over the inter-band counterpart, and in the limit Ω → 0, only the former contribution survives. Therefore, in the super-critical regime, OC displays a smooth crossover from Ω^1/*z*^ dependence (high frequency) toward a constant value as Ω → 0 (low frequency) around Ω ~ *δ*^*νz*^ [see Fig. 1]. The Drude-peak (arising from the intraband contribution) inside the metallic phase gets broadened due to a finite transport lifetime of quasiparticles, and its width increases with the strength of disorder. By contrast, inside the WSM phase and quantum critical regime the Drude-peak remains sharp.

Finally, we delve into the scaling of OC on the WSM side of the transition. In the clean limit, on dimensional grounds, we expect inter-band OC *σ*(Ω) ~ Ω^*d*−2^. Such scaling of OC remains valid in the weakly disordered WSM, at least when 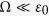, indicating that 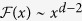 for *δ* < 0, leading to


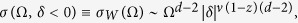


which vanishes linearly with frequency Ω. With increasing strength of disorder, the system becomes more metallic and typically at the WSM-metal QCP *z* > 1^17^,^20^,^21^,^22^,^25^,^27^,^28^,^29^,^30^,^31^. Consequently, as one approaches the WSM-metal QCP from the semimetallic side, *σ*_*W*_(Ω) increases monotonically. In the weak disorder regime, the inter-band component of OC dominates over intra-band piece until Ω ~ *E*_*F*_, with *E*_*F*_ being the Fermi energy (typically unknown) in a WSM, and with increasing frequency OC displays a smooth crossover from Ω to Ω^1/*z*^ dependence. As disorder increases the frequency range over which OC scales linearly with the frequency shrinks, while the region with Ω^1/*z*^ scaling increases. Finally, at the WSM-metal QCP *σ* ~ Ω^1/*z*^ over the entire range of frequency [see Fig. 1], at least when 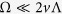.

### Optical response in a WSM

Since weak disorder flows toward smaller values with increasing RG time *l* ~ log(*v*Λ/Ω) or decreasing frequency, the lowest energy excitations are described by ballistic chiral fermions in a weakly disordered WSM. Thus, we can rely on the *Kubo formalism* in this regime to compute OC of a WSM diagramatically, and directly test the validity of its scaling ansatz for weak enough randomness. To set the stage, we first focus on the OC in a clean WSM (Δ_*N*_ = 0), which at zero temperature can be extracted from the current-current correlation function. In what follows and as shown in the SI, we compute the integrals over the internal momentum in *d* = 3 − *ε* spatial dimensions, and at the end send *ε* → 0, closely following the spirit of *dimensional regularization* that manifestly preserves the *gauge invariance*^42^,^43^. The OC in a clean WSM is 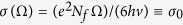, with *N*_*f*_ as the number of Weyl pairs. In this limit (Δ_*N*_ = 0), *σ*_*W*_(Ω) = *σ*(Ω), in agreement with the above scaling form. Therefore, inter-band component of OC scales *linearly* with the frequency^20^,^44^,^45^,^46^, as has been observed in Nd_2_(Ir_*x*_Rh_1−*x*_)_2_O_7_^37^ and Eu_2_Ir_2_O_7_^39^, which possibly through an “all-in all-out” magnetic ordering in pyrochlore lattice enter into a WSM phase^47^.

By now it is well established that random charge impurities (Δ_*V*_) can drive WSM-metal transition^17^,^18^,^19^,^20^,^21^,^22^,^23^,^24^,^25^,^26^,^27^,^28^,^29^,^30^,^31^,^32^ or at least can support a large crossover regime if rare regions dominate at the lowest energy scale^36^. As described in depth in the SI, elastic scatterer of any other nature (magnetic, spin-orbit, mass disorder, etc.) generates random *axial chemical potential* through quantum corrections. The axial disorder (Δ_*A*_) causes random but equal and opposite shifts of the Fermi level for left and right chiral fermions, while maintaining the overall charge neutrality of the system. Strong axial disorder also gives rise to semimetal-metal QPT^20^,^21^,^27^,^29^. Hence, to anchor the scaling behavior of OC in weak disorder regime, it is sufficient to focus on these two disorder couplings, Δ_*V*_ and Δ_*A*_, respectively characterized by two matrices 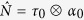 and 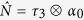. After accounting for the correction to OC to the lowest order in disorder coupling the total OC is given by (see SI for details)


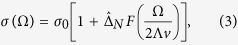


for *N* = *V*, *A*, where 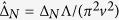 is the dimensionless bare disorder strength, and the function 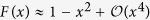. The above form of the OC 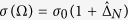 is also compatible with the scaling form of *σ*_*W*_(Ω) after substituting *z* = 3/2, *ν* = 1, as predicted from one-loop RG calculation^20^,^21^,^22^ and also reasonably consistent with recent numerical works^25^,^29^,^30^,^31^, with 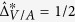 being the non-universal critical strength of disorder for the WSM-metal QPT. Such a striking agreement between scaling theory [see Eq. (2) and *σ*_*W*_(Ω)], perturbative correction to OC in the weak disorder limit [see Eq. (3)], RG and numerical analyses indicates internal consistency of our analysis, and puts forward OC as a bonafide OP across the unconventional QPT from WSM to a metallic phase. With one-loop result for the critical exponents *ν* and *z*, OC in the critical regime *σ*_*Q*_(Ω) ~ Ω^2/3^ and inside the metallic phase *σ*_*M*_(Ω → 0) ~ *δ*. However, as our scaling analysis suggests, these critical exponents can be determined independently from the scaling of OC in numerical studies and experiments to precisely determine the universality class of the WSM-metal transition. Furthermore, as shown in the SI, scaling of OC as Ω → 0 with system size (*L*) inside the metallic phase allows one to extract the correlation length exponent (*ν*) independently.

The imaginary part of OC in a weakly disordered WSM also receives a correction yielding the total dielectric constant in the presence of the chemical potential (*N* = *V*) or axial disorder (*N* = *A*)


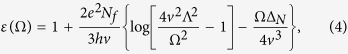


which displays a *logarithmic* enhancement as Ω → 0. It is worth mentioning that recent experiment has observed enhancement of *ε*(Ω) in Eu_2_Ir_2_O_7_ as Ω → 0^39^.

Furthermore, in the presence of arbitrary disorder the OC in three-dimensional WSM exhibits a remarkably universal dependence on frequency and disorder, but up to a sign, depending on the type of elastic scatterer, as shown in Table I of the SI. Correction to the OC due to any disorder (such as the spin-orbit one with Δ_*N*_ = Δ_*SO*_ and 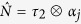, where *j* = 1, 2, 3) that together with the axial disorder also drives a WSM-metal QPT through a QCP that, however, belongs to a different universality class (with *ν* = 1, but *z* = 11/2 to one-loop order^20^,^48^), also conforms to the critical scaling form shown in Eq. (3). Furthermore, in the presence of both potential and axial disorders, WSM-metal QPT takes place through a line of QCPs in the (Δ_*V*_, Δ_*A*_) plane along which *ν* = 1 and *z* = 3/2 (to one-loop order)^20^,^48^. The OC then reads as 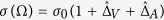, which also conforms to the universal scaling form of the OC, since the line of QCPs is given by 
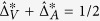
. Finally, the dielectric constant also receives a universal (up to a sign) correction due to disorder that scales linearly with frequency, as shown in Eq. (4).

## Discussion

We establish OC as an experimentally accessible OP across the disorder-driven WSM-metal QPT. In particular, we show that it can uncover signatures of an underlying dirty QCP by exposing the associated quantum critical regime at finite frequencies. While the scaling analysis is performed here strictly at *T* = 0 in the ballistic (collisionless) regime, it remains operative also at finite temperature as long as 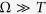
49. The finite conductivity in the metallic phase as Ω → 0 for stronger disorder should match the dc conductivity when *T* ≠ 0^50^,^51^; the value of the former is, however, expected to be different if temperature is set to be zero first. Nevertheless, irrespective of these two limits *σ*_*M*_(Ω → 0) follows the announced scaling behavior. Our scaling arguments can also be applied to the dc conductivity^20^,^22^,^26^ in the *collision-dominated* regime 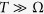, for which the scaling behavior qualitatively follows Eq. (2) upon taking Ω → *T*^49^.

Even though we primarily focused on WSMs^5^,^6^,^7^,^8^,^9^,^10^,^11^,^12^,^13^,^14^, our results are consequential to a vast number of materials, such as the topological Dirac semimetal that has recently been discovered in Cd_2_As_3_^52^ and Na_3_Bi^53^, conventional Dirac semimetals that can be found at the QCP separating two topologically distinct (for example, strong, weak, crystalline and trivial) insulating phases in various three-dimensional strong spin-orbit coupled materials, such as Bi_1−*x*_Sb_*x*_, Bi_2_Se_3_, Bi_2_Te_2_, Sb_2_Te_3_^3^,^4^ and quasi-crystals supporting Dirac fermions^38^. From the extent of the critical regime and semimetalic phase at finite frequencies (see Fig. 1), we expect that the critical scaling of OC and its correction due to random impurities can be observed in a broad class of disordered Weyl and Dirac semimetals.

## Additional Information

**How to cite this article**: Roy, B. *et al*. Universal optical conductivity of a disordered Weyl semimetal. *Sci. Rep.* **6**, 32446; doi: 10.1038/srep32446 (2016).

## Supplementary Material

Supplementary Information
