# Supplementary material for: Universal optical conductivity of a disordered Weyl semimetal
Source: Sci Rep. 2016 Aug 30;6:32446. doi: 10.1038/srep32446 (PMC5004158; doi:10.1038/srep32446)
Supplement: Supplementary Information [file srep32446-s1.pdf]

Supplementary Information for "*Universal optical conductivity of a disordered Weyl semimetal*"

Bitan Roy,[1] Vladimir Juričić,[2] and Sankar Das Sarma[1]

[1]*Condensed Matter Theory Center and Joint Quantum Institute, University of Maryland, College Park, Maryland 20742-4111, USA*

[2]*Nordita, Center for Quantum Materials, KTH Royal Institute of Technology and Stockholm University, Roslagstullsbacken 23, 10691 Stockholm, Sweden*

This Supplementary Information contains:

1. A lattice model for Weyl fermions.
2. The scaling analysis for disorder coupling in three dimensional Weyl semimetals.
3. Generation of axial disorder from arbitrary disorder through quantum loop corrections.
4. Derivation of scaling dimension of optical conductivity.
5. Calculation of optical conductivity and dielectric constant in a clean Weyl semimetal.
6. Corrections to optical conductivity and dielectric constant due to weak randomness in the system.
7. Some essential integral identities useful for the calculation of optical conductivity.
8. Derivation of the scaling of optical conductivity from renormalization group flow equations.
9. Universal scaling of finite metallic conductivity (as frequency $\Omega \to 0$) with system size (L).

• **A lattice model of Weyl semimetal.** The low energy Hamiltonian for WSM, shown in the main part of the paper, can be realized from a simple tight-binding model on a cubic lattice

$$H_W = t\sum_{\mathbf{k}} \Psi^\dagger_{\mathbf{k}} \left[\sigma_1 \cos(k_1 a) + \sigma_2 \cos(k_2 a) + \sigma_3 \cos(k_3 a) + \frac{t'}{t}\,\sigma_3 \left(\sin(k_1 a) + \sin(k_2 a) - 2\sin(k_3 a)\right)\right]\Psi_{\mathbf{k}}, \tag{1}$$

where a is the lattice spacing and $\Psi^\top_{\mathbf{k}} = [c_{\mathbf{k},\uparrow}, c_{\mathbf{k},\downarrow}]$ is a two component spinor, and $c_{\mathbf{k},\sigma}$ is the fermion annihilation operator with momentum $\mathbf{p}$ and spin projection $\alpha = \uparrow / \downarrow$. The term proportional to t' plays the role of momentum dependent *Wilson mass* that turns three pairs of Weyl nodes into massive, except the ones at $\pm\mathbf{k}_0$, respectively supporting left and right chiral fermions, where $\mathbf{k}_0 = (1,1,1)\,\frac{\pi}{2a}$. The low energy Hamiltonian in Eq. (1) of main paper is then obtained by linearizing the above tight binding model near $\pm\mathbf{k}_0$, with $v = ta/\hbar$, $\Lambda \sim 1/a$ is the ultraviolet cutoff, and momentum ($\mathbf{p}$) is measured from two Weyl nodes.

• **Scaling dimension of disorder in WSM.** The effects of disorder can be captured from the Euclidean action $S_D = \int d^3x d\tau\; V_N(\mathbf{x}) \left(\Psi^\dagger \hat{N} \Psi\right)$. We assume that all disorder assumes Gaussian white noise distribution, with disorder average $\langle\langle V_N(\mathbf{x}) V_N(\mathbf{x}')\rangle\rangle = \Delta_N \delta(\mathbf{x}-\mathbf{x}')$. Then performing the disorder averaging, we arrive at the replicated action

$$\bar{S} = \int d^d\vec{x} d\tau\; \left(\bar{\Psi}_a \left[-i\partial_\tau \gamma_0 - i\hbar v\; \gamma_j \partial_j\right]\Psi_a\right)_{(\tau,\vec{x})} - \frac{\Delta_N}{2}\int d^d\vec{x} d\tau d\tau' \left(\bar{\Psi}_a N \Psi_a\right)_{(\vec{x},\tau)} \left(\bar{\Psi}_b N \Psi_b\right)_{(\vec{x},\tau')}, \tag{2}$$

where a, b are replica indices, and $\bar{\Psi} = \Psi^\dagger \gamma_0$ as usual. For convenience we have slightly modified the definition of the γ matrices from the main part of the paper according to $\gamma_0 = \tau_1 \otimes \alpha_0$, $\gamma_j = \tau_2 \otimes \alpha_j$ for $j = 1,2,3$ and $\gamma_5 = \tau_3 \otimes \alpha_0$. These five matrices constitutes the Clifford algebra of maximal number of mutually anticommuting four dimensional matrices. Various types of elastic scatters (disorder) are realized with different choices of 4×4 matrices, as shown in Tab. I. Notice that due to linearly dispersing quasiparticles WSM corresponds to a $z = 1$ fixed point in $d = 3$. Consequently under the rescaling of space-time(imaginary) coordinates $(\mathbf{x}, \tau) \to e^l (\mathbf{x}, \tau)$. The Euclidean action $\bar{S}$ in Eq. (2) remains invariant under such coarse graining only when accompanied by rescaling of fermionic field according to $\Psi \to e^{-dl/2}\Psi$. The scaling dimension of disorder coupling then reads as $[\Delta_N] = 2z - d = -1$ for $d = 3$, for any choice of N. Therefore, any weak disorder is an irrelevant perturbation at the $z = 1$ fixed point, describing a clean

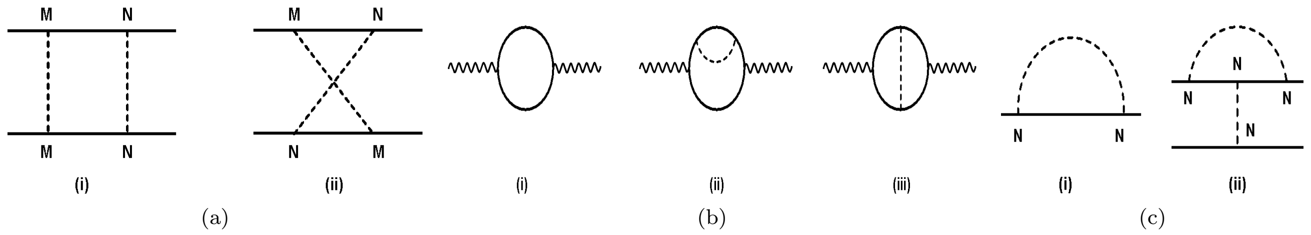

Figure 1: (a) One-loop diagrams that can generate axial disorder (Δ_A), here M and N are two 4×4 matrices. (b) OC in clean WSM arises from diagram (i), while its leading order correction due to weak disorder stems from diagrams (ii) and (iii). (c) Relevant one-loop diagrams to capture the effects of potential/axial disorder with $N = \gamma_0/\gamma_0\gamma_5$. For $M = N$, two diagrams in (a) cancel each other out. Solid, dashed and wavy lines respectively represent fermions, disorder and external gauge field.

WSM.

• **Generation of axial disorder in renormalization group framework.** We here show how axial disorder (Δ_A) can be generated from all types of disorder (except the potential disorder) once we account for quantum (loop) corrections. For this purpose computation of simple one-loop diagrams, shown in Fig. 1(a), is sufficient and serves the purpose. Total contribution from these two diagrams reads as

$$(\mathrm{a,i}) + (\mathrm{a,ii}) = \Delta_N \Delta_M \int' \frac{d^3\mathbf{k}}{(2\pi)^3} \left(\bar{\Psi}_a M G_0(0,\mathbf{k}) N \Psi_a\right) \left\{\bar{\Psi}_b \left(N G_0(0,\mathbf{k}) M + M G_0(0,-\mathbf{k}) N\right) \Psi_b\right\}, \tag{3}$$

where the integral over momentum is carried out within the Wilsonian shell $\Lambda e^{-l} < |\mathbf{k}| < \Lambda$ and $\Delta_{N/M}$ captures the strength of different types of elastic scatterer characterized by 4×4 matrix N/M (see Table I). The noninteracting Green's function is given by

$$G_0(i\omega, \mathbf{k}) = -\frac{i\gamma_0\omega + i\gamma_j k_j}{\omega^2 + k^2}. \tag{4}$$

The total contribution from these two diagrams from magnetic, spin-orbit, current and axial-magnetic impurities reads as

$$(\mathrm{a,i}) + (\mathrm{a,ii}) = \left(\Delta_M^2 + \Delta_{AM}^2 + \Delta_{SO}^2 + \Delta_C^2\right) \left(\frac{\Lambda\, l}{\pi^2 v^2}\right) \left(\bar{\Psi}_a \gamma_0\gamma_5 \Psi_a\right) \left(\bar{\Psi}_b \gamma_0\gamma_5 \Psi_b\right), \tag{5}$$

where a and b are replica indices. Thus aforementioned four disorders generate axial disorder.

The effect of mass disorder in WSM is more subtle. In Weyl semimetals the scalar (Δ_S) and pseudo-scalar (Δ_{PS}) mass disorder are present simultaneously. The quantum corrections arising from diagrams (i) and (ii) of Fig. 1(a) in the presence of two mass disorder Δ_S and Δ_{PS} yield

$$(\mathrm{a,i}) + (\mathrm{a,ii}) = \frac{\Delta_S \Delta_{PS}}{3} \left(\frac{\Lambda\, l}{\pi^2 v^2}\right) \left(\bar{\Psi}_a i\gamma_5\gamma_j \Psi_a\right) \left(\bar{\Psi}_b i\gamma_5\gamma_j \Psi_b\right), \tag{6}$$

where $j = 1,2,3$. Therefore, mass disorder (Δ_S and Δ_{PS}) in Weyl semimetal generates magnetic disorder (Δ_M) through loop corrections. As we have shown in Eq. (5), magnetic disorder in turn generates axial disorder. Therefore, to appreciate the scaling of optical conductivity it is sufficient to examine the effects of regular charge impurity (Δ_V) and axial disorder (Δ_A). A complete renormaliazation group analysis in the presence of all disorder couplings is an interesting problem, which however, falls outside the central theme of current work and deserves a separate publication [1]. Nevertheless, the above exercise justifies our claim that salient features of the scaling properties of optical conductivity can be captured by focusing on (1) random charge impurity and (2) random axial chemical potential.

• **Derivation of scaling dimension of optical conductivity.** The real part of optical conductivity at zero momentum is given in terms of the analytically continued current-current correlation function as

$$\Re\left(\sigma_{lm}\right) = \lim_{\delta \to 0} \frac{\mathrm{Im}\, \Pi_{lm}(i\Omega \to \Omega + i\delta)}{\Omega}, \tag{7}$$

Bilinear	Physical quantity	$\mathcal{T}$	$\mathcal{P}$	U_c	$\mathcal{C}$	disorder coupling	$f(\Delta_N)$
$\bar{\Psi}\gamma_0\Psi$	chemical potential	✓	✓	✓	×	Δ_V	+
$\bar{\Psi}\gamma_0\gamma_5\Psi$	axial potential	✓	×	✓	✓	Δ_A	+
$\bar{\Psi}\Psi$	scalar mass	×	✓	×	✓	Δ_S	−
$\bar{\Psi}i\gamma_5\Psi$	pseudo-scalar mass	✓	×	×	✓	Δ_{PS}	−
$\bar{\Psi}i\gamma_5\gamma_j\Psi$	magnetization	×	✓	✓	✓	Δ_M	−
$\bar{\Psi}i\gamma_j\Psi$	current	×	×	✓	×	Δ_C	−
$\bar{\Psi}\gamma_0\gamma_j\Psi$	spin-orbit coupling	×	×	×	×	Δ_{SO}	+
$\bar{\Psi}i\gamma_0\gamma_5\gamma_j\Psi$	axial-magnetization	✓	✓	×	×	Δ_{AM}	+

Table I: Various types of disorder represented by fermionic bilinears ($j=1,2,3$), their symmetries under pseudo time-reversal ($\mathcal{T}$), parity ($\mathcal{P}$), continuous chiral rotation (U_c) and charge-conjugation ($\mathcal{C}$). The disorder couplings are represented by Δ_N, and the corresponding sign function is given by $f(\Delta_N)$. Note that true time-reversal symmetry in WSM in already broken. The pseudo time-reversal symmetry $\mathcal{T}$ is generated by an anti-unitary operator $\gamma_0\gamma_2 K$, where K is complex conjugation, such that $\mathcal{T}^2=-1$ (The true time-reversal operator is $\gamma_1\gamma_3 K$). The parity operator is generated $\mathcal{P}=\gamma_0$, while the charge-conjugation operator is $\mathcal{C}=\gamma_2$. The continuous chiral symmetry (U_c) of WSM is generated by γ_5, the generator of translational symmetry in the continuum limit in clean Weyl semimetal [2]. Here ✓ and × signifies even and odd under a symmetry operation, respectively.

while the imaginary part is

$$\Im(\sigma_{lm}) = -\lim_{\delta\to 0}\frac{\mathrm{Re}\,\Pi_{lm}(i\Omega\to\Omega+i\delta)}{\Omega}. \tag{8}$$

The current-current correlation function is

$$\Pi_{lm}(i\Omega) = \int d\tau\, d^d\vec{x}\, e^{-\Omega\tau}\langle j_l(\vec{x},\tau) j_m(0,0)\rangle = \Pi(i\Omega)\delta_{lm}, \tag{9}$$

with the last equality valid in isotropic systems, which we exclusively consider here.

To find the scaling dimension of the optical conductivity, we first need to find the scaling dimension of the current. The latter is obtained from the coupling of the external vector potential to the fermions by the minimal substitution $\partial_j \to \partial_j - ieA_j$. Consequently, the gauge invariant current-gauge field coupling is

$$S_{j-A} = \int d^d\vec{x}\, d\tau\, j_i A_i. \tag{10}$$

The scaling dimension of the gauge field is $[A]=-1$ in units of the length, i.e. $[L]=1$, and therefore $[j]=-z-d+1$. Here, z is the dynamical critical exponent which determines the relative scaling between the frequency and the momentum, $\omega\sim k^z$, and therefore $[\omega]=-z$. From Eq. (9), we then obtain $[\Pi]=+d+z+2[j]=-z-d+2$. Finally, from Eq. (7) it follows that the scaling dimension of the optical conductivity is $[\sigma]=-z-d+2+z=2-d$. The scaling dimension of the optical conductivity is protected by the gauge invariance and therefore does not receive any correction, i.e. its anomalous dimension is precisely equal to zero.

• **Optical properties of a clean WSM.** We now present the calculation of the optical conductivity (OC) of a clean WSM. The OC in clean WSM at zero temperature ($T=0$) can be extracted from the current-current correlation function (see Fig. 1(b)(i) for corresponding Feynman diagram), which as a function of external frequency (Matsubara) has the form

$$\Pi^{(0)}_{lm}(i\Omega) = \frac{e^2}{h}\frac{1}{v^{d-2}}\mathrm{Tr}\int\frac{d\omega d^d\mathbf{k}}{(2\pi)^d}\gamma_l G_0(i(\omega+\Omega),\mathbf{k})\gamma_m G_0(i\omega,\mathbf{k}), \tag{11}$$

where Tr denotes the trace over the γ-matrices. Performing the above trace and integral in $d=3$, we obtain

$$\Pi^{(0)}_{lm}(i\Omega) = -\left(\frac{e^2}{h}\right)\frac{N_f\,\Omega^2}{6\pi v}\log\left[1+\frac{4v^2\Lambda^2}{\Omega^2}\right]\delta_{lm}, \tag{12}$$

where N_f is the number of Weyl pairs. After performing the *analytic continuation* to real frequency $i\Omega\to\Omega+i\delta$ (Kubo formalism), we obtain the real part of OC

$$\Re(\sigma) = \lim_{\delta\to 0}\frac{\mathrm{Im}\,\Pi(i\Omega\to\Omega+i\delta)}{\Omega} = \frac{e^2 N_f\,\Omega}{6hv}\equiv\sigma_0. \tag{13}$$

Therefore, inter-band component of OC vanishes *linearly* with the frequency (Ω), and a clean three-dimensional WSM in strict sense is a *power-law insulator.*

The imaginary part of the OC is given by

$$\Im(\sigma) = -\lim_{\delta\to 0} \frac{\mathrm{Re}\,\Pi(i\Omega \to \Omega + i\delta)}{\Omega} = -\frac{\sigma_0}{\pi} \log\left[\frac{4v^2\Lambda^2}{\Omega^2} - 1\right], \tag{14}$$

which reconciles with the result obtained by using the Kramers-Kronig (KK) relation to the real part of OC

$$\Im(\sigma) \;=\; -\frac{2\Omega}{\pi}\,\mathcal{P}\int_0^{2v\Lambda} d\Omega'\,\frac{\Re(\sigma)}{\Omega'^2 - \Omega^2}, \tag{15}$$

where $\mathcal{P}$ represents the principal value of the integral. The integral over frequency is limited by the bandwidth of a WSM $2v\Lambda$. The imaginary part of the OC is related with the real part of dielectric constant according to $\Re(\varepsilon) = 1 - 4\pi\Im(\sigma)/\Omega$. Thus in a clean WSM, the dielectric function scales as

$$\Re(\varepsilon) = 1 + \frac{e^2}{h}\,\frac{2N_f}{3v}\,\log\left[\frac{4v^2\Lambda^2}{\Omega^2} - 1\right], \tag{16}$$

which displays a *logarithmic enhancement* as frequency $\Omega \to 0$.

• **Optical conductivity of weakly disordered WSM.** We now present details of calculation for the correction to the OC in weakly disordered WSM arising from the disorder represented by the 4×4 matrix $\hat{N}$ in Table I. Corrections to current-current correlation function due to disorder stem from Feynman diagrams (ii) and (iii) in Fig. 1(b), and the total contribution from these two diagrams can be written as

$$\delta\Pi^{\hat{N}}_{lm}(i\Omega) = \Sigma^{\hat{N}}_{lm}(i\Omega) + V^{\hat{N}}_{lm}(i\Omega). \tag{17}$$

The contribution from the self-energy and vertex diagrams in Fig. 1(b) (ii) and (iii) are respectively given by

$$\Sigma^{\hat{N}}_{lm}(i\Omega) \;=\; \frac{e^2}{h}\,\frac{2}{v^{2d-2}}\Delta_N\,\mathrm{Tr}\int\frac{d^d\mathbf{k}\,d^d\mathbf{p}\,d\omega}{(2\pi)^{2d}}\,\gamma_l\,G_0(i(\omega+\Omega),\mathbf{k})\gamma_m G_0(i\omega,\mathbf{k})\hat{N}G_0(i\omega,\mathbf{p})\hat{N}G_0(i\omega,\mathbf{k}), \tag{18}$$

$$V^{\hat{N}}_{lm}(i\Omega) \;=\; \frac{e^2}{h}\,\frac{1}{v^{2d-2}}\Delta_N\,\mathrm{Tr}\int\frac{d^d\mathbf{k}\,d^d\mathbf{p}\,d\omega}{(2\pi)^{2d}}\,\gamma_l\,G_0(i(\omega+\Omega),\mathbf{k})\hat{N}G_0(i(\omega+\Omega),\mathbf{p})\gamma_m G_0(i\omega,\mathbf{p})\hat{N}G_0(i\omega,\mathbf{k}). \tag{19}$$

Different types of disorder correspond to different choices of the matrix $\hat{N}$ in the above two equations [see Table I].

We fist consider random charge impurity (with $\hat{N}=\gamma_0$) and axial potential disorder (with $\hat{N}=\gamma_0\gamma_5$) [see Table I]. For these two types of elastic scatterers contributions from Eqs. (18) and (19) after performing the trace read as

$$\Sigma^{\hat{N}=\gamma_0/\gamma_0\gamma_5}_{lm}(i\Omega) \;=\; -\delta_{lm}\frac{8N_f\Delta_N}{v^{2d-2}}\left(\frac{e^2}{h}\right)\int\frac{d^d\mathbf{k}\,d^d\mathbf{p}\,d\omega}{(2\pi)^{2d}}\,\frac{\omega(\omega+\Omega)(\omega^2-k^2) - 2(\frac{2}{d}-1)\omega^2k^2}{((\omega+\Omega)^2+k^2)(\omega^2+k^2)^2\,(\omega^2+p^2)}, \tag{20}$$

$$V^{\hat{N}=\gamma_0/\gamma_0\gamma_5}_{lm}(i\Omega) \;=\; -\delta_{lm}\frac{4N_f\Delta_N}{v^{2d-2}}\left(\frac{e^2}{h}\right)\int\frac{d^d\mathbf{k}\,d^d\mathbf{p}\,d\omega}{(2\pi)^{2d}}\,\frac{\left[\omega(\omega+\Omega)-\left(\frac{2}{d}-1\right)k^2\right]\left[\omega(\omega+\Omega)-\left(\frac{2}{d}-1\right)p^2\right]}{(\omega^2+k^2)\,(\omega^2+p^2)\,\left[(\omega+\Omega)^2+k^2\right]\,\left[(\omega+\Omega)^2+p^2\right]}, \tag{21}$$

for $N = V, A$. Upon performing the integrals, we then obtain the correction to the fermion bubble to be

$$\delta\Pi^{\hat{N}=\gamma_0/\gamma_0\gamma_5}_{lm}(i\Omega) = \delta_{lm}\,\frac{e^2N_f\Delta_N}{h}\times\frac{(i\Omega)^3}{24\pi v^4}.$$

Next we account for the scalar and pseudo scalar mass disorder, for which $\hat{N} = \hat{1}_{4\times4}$ and $i\gamma_5$, respectively, as shown in Table I, where $\hat{1}_{4\times4}$ is the 4×4 unity matrix. After performing the trace we obtain

$$\Sigma^{\hat{N}=I_{4\times4}/i\gamma_5}_{lm}(i\Omega) \;=\; -\delta_{lm}\frac{8N_f\Delta_N}{v^{2d-2}}\left(\frac{e^2}{h}\right)\int\frac{d^d\mathbf{k}\,d^d\mathbf{p}\,d\omega}{(2\pi)^{2d}}\,\frac{\omega(\omega+\Omega)(\omega^2-k^2) - 2(\frac{2}{d}-1)\omega^2k^2}{((\omega+\Omega)^2+k^2)(\omega^2+k^2)^2\,(\omega^2+p^2)}, \tag{22}$$

$$V^{\hat{N}=I_{4\times4}/i\gamma_5}_{lm}(i\Omega) \;=\; \delta_{lm}\frac{4N_f\Delta_N}{v^{2d-2}}\left(\frac{e^2}{h}\right)\int\frac{d^d\mathbf{k}\,d^d\mathbf{p}\,d\omega}{(2\pi)^{2d}}\,\frac{\left[\omega(\omega+\Omega)-\left(\frac{2}{d}-1\right)k^2\right]\left[\omega(\omega+\Omega)-\left(\frac{2}{d}-1\right)p^2\right]}{(\omega^2+k^2)\,(\omega^2+p^2)\,\left[(\omega+\Omega)^2+k^2\right]\,\left[(\omega+\Omega)^2+p^2\right]}, \tag{23}$$

for $N = S, PS$ [see Table I]. After completing the frequency and momentum integrals we obtain

$$\delta\Pi_{lm}^{\hat{N}=I_{4\times4}/i\gamma_5}(i\Omega) = -\delta_{lm}\,\frac{e^2 N_f \Delta_N}{h} \times \frac{(i\Omega)^3}{24\pi v^4}.$$

Next we focus on current disorder represented by $\hat{N} = i\gamma_j$ and random magnetic impurities for which $\hat{N} = i\gamma_j\gamma_5$, where $j = 1, 2, \cdots, d$. After performing the trace in Eqs. (18) and (19), we obtain the following contributions from the self-energy and vertex correction in the fermion bubble

$$\Sigma_{lm}^{\hat{N}=i\gamma_j/i\gamma_j\gamma_5}(i\Omega) = -\delta_{lm}\frac{8dN_f\Delta_N}{v^{2d-2}}\left(\frac{e^2}{h}\right)\int \frac{d^d\mathbf{k}\,d^d\mathbf{p}\,d\omega}{(2\pi)^{2d}}\,\frac{\omega(\omega+\Omega)(\omega^2-k^2)-2(\frac{2}{d}-1)\omega^2k^2}{((\omega+\Omega)^2+k^2)(\omega^2+k^2)^2\,(\omega^2+p^2)}, \tag{24}$$

$$V_{lm}^{\hat{N}=i\gamma_j/i\gamma_j\gamma_5}(i\Omega) = -\delta_{lm}\frac{4(2-d)N_f\Delta_N}{v^{2d-2}}\left(\frac{e^2}{h}\right)\int \frac{d^d\mathbf{k}\,d^d\mathbf{p}\,d\omega}{(2\pi)^{2d}}\,\frac{\left[\omega(\omega+\Omega)-\left(\frac{2}{d}-1\right)k^2\right]\left[\omega(\omega+\Omega)-\left(\frac{2}{d}-1\right)p^2\right]}{(\omega^2+k^2)\,(\omega^2+p^2)\,\left[(\omega+\Omega)^2+k^2\right]\,\left[(\omega+\Omega)^2+p^2\right]}, \tag{25}$$

for $N = C, M$ (see Table I). After completing the integral over frequency and momentum the correction to the polarization bubble is given by

$$\delta\Pi_{lm}^{\hat{N}=i\gamma_j/i\gamma_j\gamma_5}(i\Omega) = -\delta_{lm}\,\frac{e^2 N_f \Delta_N}{h} \times \frac{(i\Omega)^3}{24\pi v^4}.$$

Finally we delve into spin-orbit and axial magnetic disorder for which $\hat{N} = \gamma_0\gamma_j$ and $\hat{N} = i\gamma_0\gamma_j\gamma_5$, respectively, as shown in Table I. For these two choices of disorder vertex the contribution from the self-energy and vertex diagrams read as

$$\Sigma_{lm}^{\hat{N}=\gamma_0\gamma_j/i\gamma_0\gamma_j\gamma_5}(i\Omega) = -\delta_{lm}\frac{8dN_f\Delta_N}{v^{2d-2}}\left(\frac{e^2}{h}\right)\int \frac{d^d\mathbf{k}\,d^d\mathbf{p}\,d\omega}{(2\pi)^{2d}}\,\frac{\omega(\omega+\Omega)(\omega^2-k^2)-2(\frac{2}{d}-1)\omega^2k^2}{((\omega+\Omega)^2+k^2)(\omega^2+k^2)^2\,(\omega^2+p^2)}, \tag{26}$$

$$V_{lm}^{\hat{N}=\gamma_0\gamma_j/i\gamma_0\gamma_j\gamma_5}(i\Omega) = \delta_{lm}\frac{4(2-d)N_f\Delta_N}{v^{2d-2}}\left(\frac{e^2}{h}\right)\int \frac{d^d\mathbf{k}\,d^d\mathbf{p}\,d\omega}{(2\pi)^{2d}}\,\frac{\left[\omega(\omega+\Omega)-\left(\frac{2}{d}-1\right)k^2\right]\left[\omega(\omega+\Omega)-\left(\frac{2}{d}-1\right)p^2\right]}{(\omega^2+k^2)\,(\omega^2+p^2)\,\left[(\omega+\Omega)^2+k^2\right]\,\left[(\omega+\Omega)^2+p^2\right]}, \tag{27}$$

for $N = SO, AM$. Correction to the polarization bubble due to these two types of elastic scatterers is given by

$$\delta\Pi_{lm}^{\hat{N}=\gamma_0\gamma_j/i\gamma_0\gamma_j\gamma_5}(i\Omega) = \delta_{lm}\,\frac{e^2 N_f \Delta_N}{h} \times \frac{(i\Omega)^3}{24\pi v^4}.$$

From the correction to the polarization bubble due to disorder we obtain the total imaginary part of the OC to be

$$\Im(\sigma)(\Omega) = \frac{e^2}{h}\,\frac{N_f\Omega}{6\pi v}\,\left\{-\log\left[\frac{4v^2\Lambda^2}{\Omega^2}-1\right] + f\left(\Delta_N\right)\,\frac{\Omega\Delta_N}{4v^3}\right\}, \tag{28}$$

where $f(x)$ is a *sign* function that takes values ±1 depending on the nature of the impurity scatterer, as shown in Table I. The imaginary part of the OC is related to the dielectric function, which now reads as

$$\varepsilon(\Omega) = 1 - \frac{4\pi}{\Omega}\Im(\sigma)(\Omega) = 1 + \frac{2e^2N_f}{3hv}\,\left\{\log\left[\frac{4v^2\Lambda^2}{\Omega^2}-1\right] - f\left(\Delta_N\right)\,\frac{\Omega\Delta_N}{4v^3}\right\}. \tag{29}$$

By employing the second Kramers-Kronig relation we can immediately find the correction to the real part of the OC to be

$$\delta\Re(\sigma)(\Omega) = \frac{2}{\pi}\,\mathcal{P}\int_0^{2v\Lambda} d\Omega'\,\frac{\Omega'\,\delta\Im(\sigma)(\Omega')}{\Omega'^2-\Omega^2}, \quad \text{where } \delta\Im(\sigma)(\Omega) = f(\Delta_N)\,\frac{\sigma_0}{\pi}\times\frac{\Delta_N\Omega}{4v^3}, \tag{30}$$

leading to

$$\delta\Re(\sigma)(\Omega) = \sigma_0\,\left(\frac{\Delta_N\Lambda}{\pi^2v^2}\right)\,\left[1-\left(\frac{\Omega}{2v\Lambda}\right)\coth^{-1}\left(\frac{2v\Lambda}{\Omega}\right)\right]\,f\left(\Delta_N\right). \tag{31}$$

Hence the total OC in wekly disordered WSM is given by

$$\Re(\sigma)(\Omega) = \sigma_0 \left[1 + \left(\frac{\Delta_N \Lambda}{\pi^2 v^2} \right) f(\Delta_N) + f(\Delta_N) \mathcal{O}\left(\left[\frac{\Omega}{2v\Lambda} \right]^2 \right) \right]. \tag{32}$$

Notice that in three spatial dimensions $\Delta_N \Lambda/(\pi^2 v^2) = \hat{\Delta}_N$ is the dimensionless disorder coupling. Hence, to the leading order in disorder coupling and for small frequency, i.e. $\Omega/(2v\Lambda) \ll 1$, we can compactly write the total OC as

$$\Re(\sigma)(\Omega) = \sigma_0 \left[1 + f(\Delta_N) \; \hat{\Delta}_N \right]. \tag{33}$$

• **Method of integration.** Finally, we comment on some essential mathematical steps that were used to compute the optical conductivity of clean and weakly disordered WSMs. Notice that integral over internal frequency (ω) can readily be performed using *residue* formula [3]. On the other hand, we here compute the integrals over the internal momentum in spatial dimensions $d = 3 - \epsilon$, and at the end send $\epsilon \to 0$, closely following the spirit of *dimensional regularization* that manifestly preserves the *gauge invariance* [4–6].

In particular, when we compute the correction to OC due to disorder [from two-loop diagrams (ii) and (iii) in Fig. 1(b)] for which the expressions are given in Eqs. (18) and (19), the strategy is to first perform the integrals over the Matsubara frequency ω and the momentum $\mathbf{k}$, and only at the end integrate over the momentum $\mathbf{p}$. In the last step, it is useful to separately integrate out the part of the integrand that is *even* under $p \to -p$ in $d = 3$. Using the fact that this piece is of the form

$$I = \csc(d\pi) \int_0^\infty dp \, F(d,p), \tag{34}$$

where $d = 3 - \epsilon$, $\csc(x) = 1/\sin(x)$, and the function $F(d,p)$ has the property $F(d,-p) = (-1)^{2d} F(d,p)$. To compute the last integral, let us consider the following integral, which in our case can readily be computed by using the Cauchy theorem [3]

$$\int_{-\infty}^\infty dp \; \csc(d\pi) \, F(d,p) = C + \mathcal{O}(\epsilon). \tag{35}$$

After splitting this integral in two parts from $-\infty$ to 0, and 0 to ∞, substituting $p \to -p$ in the first term and using the *parity property* of the function $F(d,p)$, we have

$$\int_{-\infty}^\infty dp \csc(d\pi) F(d,p) = \left(1 + (-1)^{2d}\right) I = [2 + C_1 \epsilon + \mathcal{O}(\epsilon^2)] I, \tag{36}$$

with C_1 as a constant. We know that the left hand side is of the form $C + \mathcal{O}(\epsilon)$, and therefore

$$I = C/2 + \mathcal{O}(\epsilon). \tag{37}$$

The part that is odd under $p \to -p$ is easily computed by using some elementary integrations.

• **Derivation of scaling function for conductivity from renormalization group flow equations.** Let us now present the derivation of the scaling function of optical conductivity in the presence of potential/axial disorder couplings from the renormalization group (RG) flow equations of various coupling constants. After evaluating the relevant one-loop diagrams, shown in Fig. 1(c), we arrive at the flow equations

$$\frac{dv}{dl} = v[z - 1 - \Delta_N], \quad \frac{d\Delta_N}{dl} = -(d-2)\Delta_N + 2\Delta_N^2, \tag{38}$$

for $N = V/A$. The dimensionless disorder coupling goes as $\Delta_N \Lambda/(2\pi^2 v^2) \to \Delta_N$. Since the flow equations for these two coupling constants are identical due to the underlying chiral symmetry in WSM, from here onward we only focus only on one of these two disorder couplings and set $\Delta_N = \Delta$ for $N = V/A$. The Wilsonian momentum-shell RG scheme has previously been highlighted in this Supplementary Information. From the second equation it is clear that WSM-metal quantum phase transition takes place at a critical strength of disorder coupling (dimensionless) $\Delta_c = \frac{d-2}{2} = 1/2$ for $d = 3$. The Fermi velocity can be kept marginal during the procedure of coarse graining if we

allow a scale dependent dynamic scaling exponent $z(l) = 1 + \Delta_N(l)$. Therefore, at the WSM-metal quantum critical point $z = z_c = 3/2$ in $d = 3$ and the correlation length exponent $\nu = (d-2)^{-1}$. Since under the coarse graining the high energy cut-off $E_\Lambda = v\Lambda$ goes as $E_\Lambda \to e^{-zl} E_\Lambda$, we can arrive at the flow equation of dimensionless energy, defined as $\varepsilon = \Omega / E_\Lambda$

$$\frac{d\varepsilon}{dl} = z(l)\ \varepsilon \ \Rightarrow \ \frac{d\varepsilon}{dl} = [1 + \Delta(l)]\ \varepsilon, \tag{39}$$

where Ω is the frequency and for convenience we set $\hbar = 1$.

Since the gauge invariance mandates that the scaling dimension of conductivity is $(2-d)$, the conductivity scales as $\sigma \sim L^{2-d}$, as stated in the main part of the paper. Therefore, to extract the scaling behavior of the OC in different regimes of the phase diagram (such as WSM, quantum critical regime and metal), shown in Fig. 1 of the paper, we need to identify the appropriate RG scale that sets the *infrared* cutoff for the flow of coupling constants. The flow equations for disorder coupling Δ from Eq. (38) and dimensionless energy ε from Eq. (39) can readily be solved to obtain [7]

$$\Delta(l) = \frac{\Delta_c}{1 + \left[\frac{\Delta_c}{\Delta_0} - 1\right] e^{(d-2)l}}, \quad \varepsilon(l) = \varepsilon_0 e^{z_c l} \left(\frac{\Delta_c}{\Delta_0}\right)^{(z_c-1)\nu} \frac{1}{\left[1 + \left[\frac{\Delta_c}{\Delta_0} - 1\right] e^{(d-2)l}\right]^{(z_c-1)\nu}}, \tag{40}$$

where the quantities with subscript "0" represent their bare values. In any regime of the phase diagram, there is a competition between three length scales: spatial correlation length (ξ_l), temporal correlation length (ξ_t) and system size (L). As the critical point is approached the former two length scales respectively diverge as $\xi_l \sim |\delta|^{-\nu}$ and $\xi_t \sim |\delta|^{-z\nu}$, where $\delta = 1 - \Delta_c/\Delta_0$ measures the reduced distance from the critical point.

When $\Delta_0 > \Delta_c$ the disorder coupling in the zero energy limit ($\Omega \to 0$) diverges at the RG scale ξ_l, indicating the onset of a metallic phase at stronger disorder ($\Delta_0 > \Delta_c$). Knowing the scaling dimension of OC to be $2-d$, we can immediately arrive at the scaling of OC as $\Omega \to 0$ inside the metallic phase to be

$$\sigma_M(\Omega \to 0) \sim (\xi_l)^{2-d} \ \Rightarrow \ \sigma_M(\Omega \to 0) \sim |\delta|^{\nu(d-2)}, \tag{41}$$

in accordance with the quoted scaling form in the main part of the paper.

At the critical point ξ_l and ξ_t both diverge, and the infrared cut-off e^l for the RG flow is set by the energy at which $\varepsilon(l) \sim 1$. Hence, from the solution of $\varepsilon(l)$ we obtain $e^l \sim |\Omega|^{-1/z_c}$, when $\Delta_0 = \Delta_c$ (notice in our notation $z = z_c$ when $\Delta_0 = \Delta_c$). Therefore, the scaling of the OC at the critical point is given by

$$\sigma_Q(\Omega) \sim \left(e^l\right)^{2-d} \ \Rightarrow \ \sigma_Q(\Omega) \sim \Omega^{(d-2)/z_c}, \tag{42}$$

as $\Omega \to 0$ in agreement with the result announced in the main part of the paper. Such scaling behavior, however, persists over the entire quantum critical regime (see Fig. 1 of the paper).

In the semimetallic side of the the phase diagram $\Delta(l) \to 0$ as $l \to \infty$. Thus, in this regime we can neglect the factor of *unity* in the denominator of the second equation of Eq. (40). The relevant RG scale in this regime is again found from the condition $\varepsilon(l) \sim 1$, yielding $e^l \sim \Omega^{-1} |\delta|^{(z_c-1)\nu}$. Therefore, scaling of the OC in the WSM phase for weak disorder is given by

$$\sigma_W(\Omega) \sim \left(e^l\right)^{2-d} \ \Rightarrow \ \sigma_W(\Omega) \sim \Omega^{d-2}\ |\delta|^{(1-z_c)(d-2)\nu}, \tag{43}$$

which is also in agreement with the predicted scaling form of OC inside the semimetallic regime of the phase diagram of a dirty WSM. Therefore, from the solution of leading order RG flow equations and proper identification of the infrared curt-off for the RG flow, we can arrive at the leading order scaling behavior of the OC inside the metallic, quantum critical and Weyl semimetal phases.

Within this framework, one can immediately arrive at the scaling behavior (at least the leading oder) of dc conductivity (in the collision dominated regime), simply by redefining the dimensionless energy scales to be $\varepsilon = T/E_0$ (after setting $k_B = 1$). Thus, we strongly believe that scaling behavior for both ac and dc conductivity are

qualitatively the same in dirty WSMs.

• **Scaling of finite metallic conductivity (as frequency $\Omega \to 0$) with system size.** As shown in the main part of the paper the scaling behavior of optical conductivity is captured by the following universal scaling ansatz

$$\sigma(\Omega, \delta, L) = L^{2-d} \mathcal{G}\left(\frac{L}{\delta^{-\nu}}, \frac{\Omega}{\delta^{\nu z}}\right) = \delta^{\nu(d-2)} \mathcal{F}\left(\frac{L}{\delta^{-\nu}}, \frac{\Omega}{\delta^{\nu z}}\right), \tag{44}$$

where $\mathcal{G}$ and $\mathcal{F}$ are two unknown, but universal scaling functions. We now focus on the scaling of the finite metallic conductivity as $\Omega \to 0$ inside the compressible diffusive metallic phase when $\delta > 0$ or $\Delta_N > \Delta_N^*$. Setting $\Omega = 0$ in the above equation we obtain

$$\sigma(0, \delta, L) = \delta^{\nu(d-2)} \mathcal{F}\left(L\delta^{\nu}, 0\right). \tag{45}$$

Thus upon extracting the finite metallic conductivity for various system sizes and strengths of strong disorder couplings (i.e. when $\delta > 0$ or $\Delta_N > \Delta_N^*$) one can independently extract the correlation length exponent (ν) across the WSM-metal quantum phase transition from the data collapse obtained by comparing $\sigma(0, \delta, L)\delta^{\nu(2-d)}$ vs. $L\delta^{\nu}$ or $L^{1/\nu}\delta$.

[1] B. Roy, and V. Juričić, *Classification of disorder driven Weyl semimetal-metal quantum phase transitions in three dimensions*, Unpublished.
[2] B. Roy, and J. D. Sau, *Magnetic catalysis and axionic charge-density-wave in Weyl semimetals*, Phys. Rev. B **92**, 125141 (2015).
[3] G. B. Arfken, H. J. Weber, and F. E. Harris, *Mathematical Methods for Physicists: A Comprehensive Guide* (Academic Press, 7th ed., 2012).
[4] G. 't Hooft and M. J. T. Veltman, *Regularization and renormalization of gauge fields*, Nucl. Phys. B **44**, 189 (1972).
[5] M. E. Peskin and D. V. Schroeder, *An Introduction to Quantum Field Theory* (Addison-Wesley, Reading, MA, 1995).
[6] V. Juricic, O. Vafek, I. F. Herbut, *Conductivity of interacting massless Dirac particles in graphene: Collisionless regime*, Phys.Rev.B **82**, 235402 (2010).
[7] J. H. Pixley, P. Goswami, and S. Das Sarma, *Disorder-driven itinerant quantum criticality of three-dimensional massless Dirac fermions*, Phys. Rev. B **93**, 085103 (2016).
